# Association of depression and diabetes complications and mortality: a population-based cohort study

**DOI:** 10.1017/S2045796020000049

**Published:** 2020-01-29

**Authors:** C.-S. Wu, L.-Y. Hsu, S.-H. Wang

**Affiliations:** 1 Department of Psychiatry, National Taiwan University Hospital, Taipei, Taiwan; 2 College of Medicine, National Taiwan University, Taipei, Taiwan; 3 Institute of Epidemiology and Preventive Medicine, College of Public Health, National Taiwan University, Taipei, Taiwan; 4 Department of Occupational Safety and Health, China Medical University, Taichung, Taiwan; 5 Department of Public Health, China Medical University, Taichung, Taiwan

**Keywords:** Advanced complications, depression, diabetes, macrovascular, microvascular, mortality

## Abstract

**Aims:**

Several studies suggested that depression might worsen the clinical outcome of diabetes mellitus; however, such association was confounded by duration of illness and baseline complications. This study aimed to assess whether depression increases the risk of diabetes complications and mortality among incident patients with diabetes.

**Methods:**

This was a population-based matched cohort study using Taiwan's National Health Insurance Research Database. A total of 38 537 incident patients with diabetes who had depressive disorders and 154 148 incident diabetes patients without depression who were matched by age, sex and cohort entry year were randomly selected. The study endpoint was the development of macrovascular and microvascular complications, all-cause mortality and cause-specific mortality.

**Results:**

Among participants, the mean (±SD) age was 52.61 (±12.45) years, and 39.63% were male. The average duration of follow-up for mortality was 5.5 years, ranging from 0 to 14 years. The adjusted hazard ratios were 1.35 (95% confidence interval [CI], 1.32–1.37) for macrovascular complications and 1.08 (95% CI, 1.04–1.12) for all-cause mortality. However, there was no association of depression with microvascular complications, mortality due to cardiovascular diseases or mortality due to diabetes mellitus. The effect of depression on diabetes complications and mortality was more prominent among young adults than among middle-aged and older adults.

**Conclusions:**

Depression was associated with macrovascular complications and all-cause mortality in our patient cohort. However, the magnitude of association was less than that in previous studies. Further research should focus on the benefits and risks of treatment for depression on diabetes outcome.

## Introduction

Diabetes mellitus is one of the leading causes of mortality and causes severe complications, including stroke, myocardial infarction, chronic kidney disease and amputation due to diabetes foot (Stratton *et al*., 2000). Depression is also associated with increased mortality (Walker *et al*., 2015) and medical complications (Katon, 2003). Previous studies have demonstrated bidirectional associations between the incidence of depression and diabetes mellitus (Pan *et al*., 2010; Chen *et al*., 2013). Depressive disorders increase the risk of new-onset diabetes mellitus. However, patients with diabetes mellitus also have higher likelihood of developing depressive symptoms. Furthermore, depression is associated with poor prognosis of cardiovascular diseases, cancer and surgical procedures (Pinquart and Duberstein, 2010; Hare *et al*., 2013; Ghoneim and O'Hara, 2016).

Several studies have demonstrated that in patients with diabetes, depressive disorders are associated with poor glycemic control, although with a small effect size (Lustman *et al*., 2000). One meta-analysis demonstrated an association between depression and diabetes complications (De Groot *et al*., 2001); however, most included studies were cross-sectional, small-scale studies. Recently, several prospective studies have focused on the effects of depression on the risk of diabetes complications (Black *et al*., 2003; Orchard *et al*., 2003; Bruce *et al*., 2005; Egede *et al*., 2005; Katon *et al*., 2005; Richardson *et al*., 2008; Lin *et al*., 2010; Mai *et al*., 2011; Pan *et al*., 2011; Scherrer *et al*., 2011; Sullivan *et al*., 2012; Ting *et al*., 2013; Ismail *et al*., 2017). The results have been inconsistent. Most studies showed that baseline depressive symptoms or disorders were associated with increased risk of diabetes complications (Black *et al*., 2003; Orchard *et al*., 2003; Lin *et al*., 2010; Mai *et al*., 2011; Scherrer *et al*., 2011; Ting *et al*., 2013) or mortality (Egede *et al*., 2005; Katon *et al*., 2005; Mai *et al*., 2011; Pan *et al*., 2011; Scherrer *et al*., 2011). Nevertheless, some studies showed a null association between depression and advanced microvascular complications (Sullivan *et al*., 2012; Ismail *et al*., 2017). Another study found that the association of depression with mortality might be confounded by underlying diabetes complications (Bruce *et al*., 2005). It should be noted that patients with depression have a longer duration of diabetes (Egede and Zheng, 2003) and a greater number of diabetes complications (Deschênes *et al*., 2017). Most studies have included prevalent patients with diabetes; therefore, the temporal relationship between depression and diabetes complications is unclear. A recent review showed that a substantial proportion of studies failed to demonstrate that the outcome of interest was not present at baseline (Nouwen *et al*., 2019). Only one study enrolled patients recently diagnosed with diabetes (Ismail *et al*., 2017); the findings showed that depression was only associated with macrovascular complications but not with worse glycemic control or increased risk of microvascular complications. However, the sample size in that study was moderate and the follow-up period was only 2 years.

In the present study, we used a nationally representative claims database to conduct a prospective matched cohort analysis to explore the association of depressive disorders with diabetes complications and related mortality. To eliminate confounding by the duration of illness and baseline complications, we only included incident patients with diabetes.

## Method

### Data source

In this study, we used Taiwan's National Health Insurance Research Database (NHIRD) (Bellows *et al*., 2014). Taiwan launched its universal National Health Insurance (NHI) program on March 1, 1995, which covers 99% of the Taiwanese population. The NHIRD is derived from the original reimbursement claims and all identifiable personal information is anonymized. The database includes patients' demographic variables, clinical diagnoses, medical procedures and prescription records in outpatient, emergency department and inpatient settings. The accuracy of clinical diagnoses, such as depression, diabetes, stroke and acute coronary syndrome, has been well established (Lin *et al*., 2005; Wu *et al*., 2010; 2020; Cheng *et al*., 2011). The NHIRD has been widely used in psychiatric epidemiological studies and diabetes outcome research. This study was approved by the Internal Review Board of Human Studies of China Medical University Hospital.

### Study population

Using the NHIRD, we identified incident patients with diabetes aged 20 years or more who were firstly prescribed antidiabetic agents and had at least a diagnosis of diabetes mellitus (International Classification of Diseases, Ninth Revision, Clinical Modification (ICD-9-CM) code: 250.x) between 2001 and 2014 (*n* = 1 864 089). Patients with type 1 diabetes mellitus were identified if their ICD-9 diagnostic code was 250.x1 or 250.x3 and they never used oral antidiabetic drug; the patients who remained were categorised into type 2 diabetes mellitus. The first date of antidiabetic prescription was defined as the cohort entry date. Given that some patients delayed treatment and had complications when diabetes was diagnosed, we excluded those with macro- or microvascular complications before or on the cohort entry date (*n* = 758 041). We did not include patients with a diagnosis of schizophrenia or bipolar disorders (*n* = 5664). In addition, those with information errors regarding sex, age or mortality date were also excluded (*n* = 13 259). The study population included 1 087 125 incident patients with diabetes.

### Cohort of patients with diabetes who had depressive disorders

Incident patients with depressive disorder were those with at least two or more records with diagnosis of depressive disorders before the cohort entry date. The diagnostic code included major depressive disorder, recurrent episode (ICD-9 code: 296.3x); major depressive disorder, single episode (ICD-9 code: 296.2x); dysthymic disorder (ICD-9 code: 300.4) or depressive disorder not otherwise specified (ICD-9 code: 311). If a patient has two or more different diagnostic codes of depression, the types of depression were categorised based on the above-mentioned hierarchy. Finally, we identified a total of 38 537 patients with diabetes who had depressive disorders.

### Comparison cohort of patients who had diabetes with no depressive disorders

**Fig. 1.**
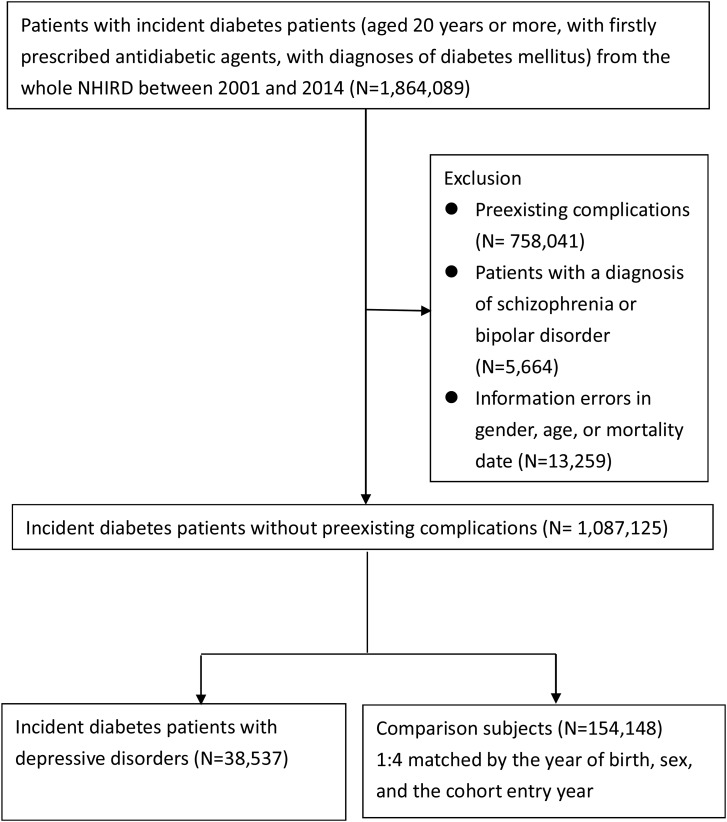
Flow chart of selection of study population.

We identified 154 148 eligible comparison participants with diabetes mellitus, who had no preexisting complications and no diagnosis of severe mental illnesses. For each patient with diabetes who had a depressive disorder, we randomly selected four comparison participants matched by age (year of birth), sex and the cohort entry year. The comparison participants were censored if they were diagnosed with a depressive disorder after the cohort entry date. The details of the selection procedure are shown in Fig. 1.

### Main outcome measures

The study outcomes included macro- and microvascular diabetes complications, which were identified based on ICD-9-CM diagnostic and procedural codes and the NHI procedural codes from outpatient and inpatient claims records. Macrovascular complications included hospitalisation for acute coronary syndrome (ICD-9-CM: 410.x-411.x) and stroke (ICD-9-CM: 430.x-438.x). Validation of the inpatient diagnosis of the acute coronary syndrome and stroke has been well documented (Wu *et al*., 2010; Cheng *et al*., 2011). Nevertheless, the accuracy of the diagnostic codes of microvascular complications remains unclear. Thus, we used a restricted definition, combining ambulatory diagnostic claims with procedural codes, or we used inpatient claims only, to ensure the accuracy of the diagnosis of microvascular complications. Microvascular complication was defined as the composite of diabetic retinopathy (ICD-9-CM: 250.5x, 362.01 or 362.02) with laser photocoagulation (NHI procedural code: 86 206B or 86207B) or vitrectomy (NHI procedural code: 60003C or 60004C), blindness (ICD-9-CM: 369.x), end-stage chronic kidney disease (ICD-9-CM: 585.5- 585.6 or 586) with dialysis, vessel operations for hemodialysis or kidney transplantation (ICD-9-CM: 39.27, 39.42, 39.43, 39.49, 39.50, 39.53, 39.93, 39.94, 39.95, 54.98 or 55.6x), hospitalisation for diabetic foot infection (ICD-9-CM: 681.1x, 682.6 or 682.7) or lower extremity amputation (ICD-9-CM procedural code: 84.1x).

Mortality information was obtained using Taiwan's National Death Registry. The cause-specific mortality included mortality due to cardiovascular disease (ICD-9: 390–459, ICD Tenth Revision (ICD-10): I00–I99) or diabetes (ICD-9: 250, ICD-10: E10-E14). To test the sensitivity of our methods, we included unnatural death (ICD-9: E800-E969, ICD-10: V, W, X or Y) and suicide (ICD-9: E950-E959, ICD-10: X60-X84 or Y87.0).

### Covariate assessment

Patients' demographic variables included age, sex and the year of cohort entry. The duration between the first diagnosis of diabetes and the initiation of pharmacotherapy was also measured and categorised into <1 and ⩾1 year. Potential confounders, which were associated with both diabetes complications and depressive disorders, were assessed in the year preceding the cohort entry date, which included comorbid conditions of hypertension (ICD-9 code: 401.x-405.x), dyslipidemia (ICD-9 code: 272.x), chronic pulmonary disease (ICD-9 code: 491.x-494.x, 496.x), chronic liver disease (ICD-9 code: 517.2x, 517.5x, 517.6x, 517.8x, 517.9x, A347.x, 572.2x, 572.3x, 572.4x, 456.0x, 456.1x or 456.2x) and alcohol- and substance-related disorders (ICD-9 code: 291.x, 292.x, 303.x, 304.x, 305.x (except 305.1). In addition, medication use that reflected underlying medical conditions was assessed, including angiotensin-converting enzyme inhibitor (ACEI) and angiotensin receptor blocker (ARB) (ATC code: C09), beta blockers (ATC code: C07), calcium channel blockers (ATC code: C08), lipid-lowering agents (ATC code: C10) and nonsteroidal anti-inflammatory drugs (ATC code: M10).

### Statistical analysis

We calculated the incidence of macro- and microvascular diabetes complications and mortality rates based on the event number divided by follow-up person-years. Multivariate Cox proportional hazards models were used with adjustment for the abovementioned confounders, to estimate the hazard ratios (HRs) of depressive disorders for the study outcomes. Subgroup analyses were conducted to evaluate whether the risks of depression for diabetes complications and mortality were modified by types of depression, age, sex and duration between diabetes diagnosis and initiation of pharmacotherapy.

The quality of diabetes care might be a mediator in the association of depression with diabetes complications and mortality; therefore, we did not include these variables in our model. We used *post-hoc* analyses to evaluate whether there were differences in the quality of diabetes care during the follow-up period between patients who had diabetes, with and without depressive disorder. Indicators of care quality included medication adherence for antidiabetic drugs, frequency of receiving blood sugar testing (HbA1c or fasting blood sugar), lipid profiles, serum creatinine, retina examinations and electrocardiograms during the follow-up period. Antidiabetic drug adherence was measured using the medication possession ratio (MPR), which was defined as the total days of prescribed antidiabetic medication supply divided by the follow-up period. Antidiabetic drug adherence was categorised as poor, irregular and good, based on the MPR (<0.2, 0.2–0.8 and ⩾0.8, respectively).

All statistical analyses were conducted using SAS version 9.4 (SAS Institute Inc., Cary, NC, USA). The statistical significance of relationships was assessed using 95% confidence intervals (CI) or *p* values <0.05.

## Results

There were 38 537 patients with diabetes who had depressive disorders and 154 148 patients with diabetes with no depressive disorders, as comparison participants. The average follow-up period was 5.5 years (ranging from 0 to 14 years). The mean (±SD) age of participants was 52.61 (±12.45) years, and 39.63% were male. Most of the patients have type 2 diabetes mellitus 99.4%. Among patients with depressive disorders, 26.21% had major depressive disorder, recurrent episode, 15.96% had major depressive disorder, single episode; 48.11% had dysthymia and 9.72% had depressive disorder, not otherwhere classified. Patients with diabetes who had depressive disorders had a higher proportion of comorbid conditions and medication use, except for ACEI/ARB use. The percentage of duration between diabetes diagnosis and initiation of pharmacotherapy ⩾1 year was 17% for patients with diabetes and depression and 24% for comparison subjects (see Table 1).

**Table 1.** Baseline characteristics (the year before entry)

	Diabetes and depression group	Comparison group	*p*-Value
	(*n* = 38 537)	(*n* = 154 148)	
Sex	15 273 (39.63)	61 092 (39.63)	NA
Age			
20–44	10 395 (26.97)	41 580 (26.97)	NA
45–64	21 434 (55.62)	85 736 (55.62)	
≧65	6708 (17.41)	26 832 (17.41)	
Type of diabetes mellitus			
Type I	22 (0.06)	85 (0.06)	0.885
Type II	38 515 (99.94)	154 063 (99.94)	
Duration between diagnosis and initiation of antidiabetic drug			
<1 year	29 120 (75.56)	127 436 (82.67)	<0.001
⩾1 year	9417 (24.44)	26 712 (17.33)	
Types of depression			
Major depressive disorder, recurrent episode	10 102 (26.21)		NA
Major depressive disorder, single episode	6149 (15.96)		
Dysthymia	18 539 (48.11)		
Depressive disorder, not otherwise classified	3747 (9.72)		
Comorbidity			
Hypertension	16 363 (42.46)	62 875 (40.79)	<0.001
Dyslipidemia	14 648 (38.01)	55 222 (35.82)	<0.001
Chronic liver diseases	2793 (7.25)	6458 (4.19)	<0.001
Chronic pulmonary diseases	4442 (11.53)	11 605 (7.53)	<0.001
Alcohol/substance use disorder	2557 (6.64)	2353 (1.53)	<0.001
Medication use			
ACEI/ARB	2214 (5.75)	8931 (5.79)	0.714
Beta blocker	2613 (6.78)	6535 (4.24)	<0.001
Calcium channel blocker	2206 (5.72)	7701 (5.00)	<0.001
Diuretics	2008 (5.21)	5068 (3.29)	<0.001
Lipid-lowering agent	4486 (11.64)	15 219 (9.87)	<0.001
NSAID	5140 (13.34)	19 863 (12.89)	0.018

ACEI/ARB, angiotensin-converting enzyme inhibitor/angiotensin receptor blocker; NSAID, nonsteroidal anti-inflammatory drugs.

The crude incidence of macrovascular complications was 74.65 per 1000 person-years for patients with diabetes and depressive disorders; this was higher than the incidence (54.65 per 1000 person-years) for comparison participants. The same was true for the crude incidence of mortality due to cardiovascular diseases (2.58 *v.* 2.29 per 1000 person-years), unnatural mortality (2.46 *v.* 0.77 per 1000 person-years), suicide (1.41 *v.* 0.27 per 1000 persona-years) and all-cause mortality (21.91 *v.* 15.96 per 1000 person-years) between patients who had diabetes, with and without depressive disorders. However, there was no difference with respect to microvascular complications and mortality due to diabetes mellitus (see Table 2).

**Table 2.** Incidence of macro, micro, diabetes-related, circulation and all-cause mortality of the diabetes & depression group and comparison group

	Macro complication	Micro complication	DM-related death	Circulation death	Unnatural death	Suicide	All-cause mortality
Diabetes & depression group (*n* = 38 537)							
Event/person-years	11 752/157 415	8239/178 982	422/211 388	546/211 388	521/211 388	297/211 388	4632/211 388
Incidence per 1000 PY (95% CI)	74.65 (73.32–76.02)	46.03 (45.05–47.04)	2.00 (1.81–2.20)	2.58 (2.38–2.81)	2.46 (2.26–2.69)	1.41 (1.25–1.57)	21.91 (21.29–22.55)
Comparison group (*n* = 154 148)							
Event/person-years	37 517/686 513	32 676/716 948	1644/842 948	1928/842 948	649/842 948	226/842 948	13 453/842 948
Incidence per 1000 PY (95% CI)	54.65 (54.10–55.21)	45.57 (45.09–46.07)	1.95 (1.86–2.05)	2.29 (2.19–2.39)	0.77 (0.71–0.83)	0.27 (0.24–0.31)	15.96 (15.69–16.23)

After adjusting for abovementioned covariates, we found that patients with diabetes who had depressive disorders had higher HRs for developing macrovascular complications (HR = 1.35; 95% CI 1.32–1.37), unnatural mortality (HR = 2.59; 95% CI 2.30–2.91), suicide (HR = 5.64; 95% CI 4.70–6.77) and all-cause mortality (HR = 1.08; 95% CI 1.04–1.12) than comparison participants. However, there was no statistically significant difference in microvascular complications and death owing to cardiovascular diseases. We found that depression was associated with a reduced risk of death owing to diabetes (Table 3).

**Table 3.** HRs for diabetes complications and mortality of the diabetes patients with and without depressive disorder, total and stratified by types of depression, gender, age and duration between diagnosis and initiation of pharmacotherapy

	Macro complication aHR (95% CI)	Micro complication aHR (95% CI)	DM-related death aHR (95% CI)	Circulation death aHR (95% CI)	Unnatural death aHR (95% CI)	Suicide aHR (95% CI)	All-cause mortality aHR (95% CI)
All samples	1.35 (1.32–1.37)	0.99 (0.97–1.02)	0.86 (0.77–0.95)	0.96 (0.88–1.06)	2.59 (2.30–2.91)	5.64 (4.70–6.77)	1.08 (1.04–1.12)
Types of depression							
MDD, recurrent	1.44 (1.39–1.50)	1.04 (0.99–1.09)	1.08 (0.87–1.33)	1.07 (0.88–1.30)	4.52 (3.64–5.63)	12.00 (8.31–17.31)	1.19 (1.11–1.28)
MDD, single	1.38 (1.31–1.45)	1.01 (0.95–1.07)	0.99 (0.75–1.30)	1.07 (0.85–1.35)	3.10 (2.31–4.16)	6.90 (4.43–10.73)	1.18 (1.09–1.28)
Dysthymia	1.31 (1.27–1.35)	0.97 (0.93–1.00)	0.71 (0.61–0.84)	0.91 (0.79–1.04)	1.89 (1.58–2.27)	3.26 (2.49–4.27)	1.01 (0.96–1.06)
Depressive disorder, NOS	1.25 (1.17–1.34)	0.97 (0.90–1.05)	0.92 (0.66–1.29)	0.85 (0.63–1.15)	1.29 (0.82–2.02)	4.80 (2.31–9.94)	1.03 (0.92–1.14)
Sex							
Male	1.30 (1.26–1.34)	0.99 (0.96–1.03)	0.87 (0.75–1.02)	0.94 (0.82–1.07)	2.24 (1.92–2.62)	4.76 (3.77–6.00)	1.06 (1.01–1.11)
Female	1.38 (1.34–1.42)	0.99 (0.96–1.02)	0.85 (0.73–0.99)	1.00 (0.87–1.14)	3.23 (2.67–3.90)	7.38 (5.48–9.95)	1.11 (1.06–1.17)
Sex × depression interaction	*p*-Value = 0.0017	*p*-Value = 0.9997	*p*-Value = 0.7069	*p*-Value = 0.4888	*p*-Value = 0.0015	*p*-Value = 0.0118	*p*-Value = 0.1745
Age							
20–44	1.51 (1.44–1.59)	1.06 (1.01–1.11)	1.22 (0.92–1.61)	1.34 (1.00–1.81)	4.00 (3.15–5.08)	8.92 (6.19–12.85)	1.35 (1.23–1.48)
45–64	1.35 (1.31–1.39)	0.98 (0.95–1.01)	0.83 (0.70–0.99)	0.92 (0.78–1.08)	2.75 (2.33–3.25)	5.29 (4.13–6.79)	1.09 (1.03–1.15)
**⩾**65	1.24 (1.19–1.29)	0.96 (0.91–1.01)	0.76 (0.64–0.90)	0.93 (0.81–1.05)	1.35 (1.04–1.76)	3.33 (2.20–5.05)	0.97 (0.92–1.02)
Age × depression interaction (45–64 *v.* 20–44) (**⩾**65 *v.* 20–44)	*p*-Value <0.0001 *p*-Value <0.0001	*p*-Value = 0.0003 *p*-Value = 0.0005	*p*-Value = 0.0002 *p*-Value <0.0001	*p*-Value = 0.0181 *p*-Value = 0.0079	*p*-Value = 0.0017 *p*-Value <0.0001	*p*-Value = 0.0070 *p*-Value = 0.0003	*p*-Value <0.0001 *p*-Value <0.0001
Duration between diagnosis and initiation of pharmacotherapy							
<1 year	1.34 (1.31–1.37)	0.97 (0.95–1.00)	0.81 (0.72–0.92)	0.92 (0.83–1.03)	0.92 (0.83–1.03)	5.45 (4.48–6.62)	1.05 (1.01–1.09)
≧1 year	1.36 (1.30–1.42)	1.06 (1.01–1.12)	1.07 (0.84–1.36)	1.16 (0.94–1.45)	1.16 (0.94–1.45)	7.06 (4.22–11.79)	1.20 (1.11–1.29)
Duration × depression interaction	*p*-Value = 0.7812	*p*-Value = 0.0013	*p*-Value = 0.0218	*p*-Value = 0.0839	*p*-Value = 0.5794	*p*-Value = 0.4134	*p*-Value = 0.0046

MDD, major depressive disorder; NOS, not otherwise specified.

In subgroup analyses, we found that the magnitude of associations with developing macrovascular complications, unnatural death, suicide and all-cause mortality was higher among those with major depression with recurrent episode than those with dysthymia or depressive disorder, not otherwise specified (the 95% CIs were not overlapping). In terms of the modifying effect of sex, we found that the associations between depression and developing macrovascular complications and unnatural mortality were stronger among women than among men. Otherwise, sex did not have a modifying effect on the incidence of microvascular complications and all-cause and other cause-specific mortality. In terms of age, there were effect modifications on diabetes complications and mortality. HRs in young adults (aged 20–44 years) with depressive disorders for all diabetes complications, all-cause mortality and all cause-specific mortality were higher than those among middle-aged (aged 45–64 years) and older (aged 65 years or more) adults. Furthermore, we found that duration between diabetes diagnosis and initiation of pharmacotherapy had a significant modifying effect on the associations with microvascular complications, diabetes-related death and all cause-mortality; the associations were stronger among patients with duration ⩾1 year than those with duration >1 year.

Assessment of the quality of diabetes care showed that patients with diabetes who had depressive disorders had slightly higher proportions of good antidiabetic compliance and higher rates of undergoing screening tests; however, the difference was quite small (Table 4).

**Table 4.** The quality of diabetes care among the diabetes patients with and without major depressive disorder

	Diabetes & depression group (*n* = 38 537) *n* (%)	Comparison group (*n* = 154 148) *n* (%)	*p*-Value of *χ*^2^ test
Fasting blood glucose/haemoglobin A1c	33 859 (87.86)	132 919 (86.23)	<0.0001
Lipid profiles	27 192 (70.56)	105 736 (68.59)	<0.0001
Urine protein profiles	22 165 (57.52)	80 209 (52.03)	<0.0001
Serum creatinine	28 844 (74.85)	105 198 (68.24)	<0.0001
Retina examination	5814 (15.09)	23 606 (15.31)	0.2677
Electrocardiogram	10 547 (27.37)	30 687 (19.91)	<0.0001
Antidiabetic drug adherence			<0.0001
MPR <0.2	13 669 (35.47)	49 224 (31.93)	
MPR 0.2–0.8	22 036 (57.18)	94 630 (61.39)	
MPR **⩾**0.8	2832 (7.35)	10 294 (6.68)	

MPR, medication possession ratio.

## Discussion

### Main findings

In this study, we found that depression was associated with an increased risk of macrovascular complications, unnatural mortality, suicide and all-cause mortality among patients with incident diabetes mellitus. In contrast, there was no association between microvascular complications and mortality owing to diabetes mellitus and cardiovascular diseases. We found that age had a multiplicative effect modification on such associations. The magnitude of association was higher among young adults than middle-aged and older adults. Regarding the quality of care, we found that patients with diabetes who had depressive disorders had slightly better antidiabetic medication compliance and a slightly higher proportion of undergoing screening tests.

### Comparison with other studies

The findings of a positive association between depression and macrovascular complications were consistent with those of several previous studies (Black *et al*., 2003; Lin *et al*., 2010; Mai *et al*., 2011; Scherrer *et al*., 2011; Ting *et al*., 2013; Ismail *et al*., 2017; Nouwen *et al*., 2019). The underlying mechanism might be multifactorial. In patients with depressive disorder, associations were found between unhealthy lifestyle behaviours, such as tobacco smoking, physical inactivity or unhealthy diet (Deschênes *et al*., 2017). Although we could not directly measure lifestyle behaviours in this study, we found that patients with depression had a higher prevalence of dyslipidemia, alcohol- or substance-related disorders and chronic pulmonary diseases, which are highly related to an unhealthy lifestyle. These unhealthy lifestyle behaviours are important contributors to macrovascular complications in patients with diabetes who have depression. In addition, depression itself might directly increase the risk of cardiovascular diseases (Joynt *et al*., 2003). Depression is linked to dysregulation of the hypothalamic–pituitary–adrenal axis, cardiac rhythm disturbances and systemic inflammation, which are also related to cardiovascular diseases (Joynt *et al*., 2003; Pariante, 2003).

We also found that depression was related to an increased all-cause mortality rate; however, the magnitude was smaller than that found in previous studies (Black *et al*., 2003; Egede *et al*., 2005; Katon *et al*., 2005; Mai *et al*., 2011; Pan *et al*., 2011; Scherrer *et al*., 2011; Winkley *et al*., 2012). In addition, we did not find an association of depression with death owing to cardiovascular diseases or diabetes mellitus. This finding was in contrast to those of earlier studies (Mai *et al*., 2011; Pan *et al*., 2011). Most previous studies included prevalent patients with diabetes who had a duration of illness of 5 years or more (Orchard *et al*., 2003; Egede *et al*., 2005; Richardson *et al*., 2008; Lin *et al*., 2010; Pan *et al*., 2011; Sullivan *et al*., 2012; Ting *et al*., 2013); patients with depression might have had a longer duration of illness and more diabetes complications at baseline (Egede and Zheng, 2003). The associations between depression and mortality among patients with diabetes might be confounded by the duration of illness and pre-existing complications. One prospective study found that the effect of depression on mortality was no longer statistically significant after adjusting for macro- and microvascular complications (Bruce *et al*., 2005). In the present study, we included patients with incident diabetes mellitus and we excluded those who had diabetes complications. In addition, our study design eliminated confounding by the duration of illness and underlying complications. Furthermore, the average duration of diabetes was 5.5 years; therefore, the case number of death owing to cardiovascular diseases or diabetes mellitus might be small to have enough statistical power. Moreover, patients with untreated depression would be misclassified into comparison groups. It would introduce a bias towards the null. However, the bias would be small due to the low prevalence of depression (<2%) in Taiwan (Liao *et al*., 2012).

We found there was no association between depression and microvascular complications. This finding was against several studies (Black *et al*., 2003; Lin *et al*., 2010) and the recent meta-analysis (Nouwen *et al*., 2019). Our result was compatible with that in a study conducted in the United Kingdom, which only included patients with a recent (<6 months) diagnosis of diabetes mellitus (Ismail *et al*., 2017); in that study, depression was not found to be associated with microvascular complications during the first 2 years of follow-up. Our study findings, with an average 5.5-year follow-up period (ranging from 0 to 14 years), further support the previous results. Glycemic control is a key factor related to the development of microvascular complications (UK Prospective Diabetes Study (UKPDS) Group, 1998). Whether patients with depressive disorders have poorer glycemic control remains inconclusive. One meta-analysis demonstrated that patients with diabetes and depression have poor glycemic control; nevertheless, the effect size was small (<0.2) (Lustman *et al*., 2000). Several recent prospective studies have shown that patients with diabetes and depression have sugar levels that are comparable to those without depression (Bruce *et al*., 2005; Ismail *et al*., 2017). Although the status of glycemic control was not available in our study database, the indicators for quality of diabetes care, including medication adherence for antidiabetic agents and frequency of screening tests, were approximately equal between patients with and without depression. Thus, we believe that there is no marked difference in glycemic control, and therefore, in the risk of microvascular complications between these patient groups. The role of glycemic control is less influential in macrovascular complications (Skyler *et al*., 2009). Unhealthy lifestyle, hypertension, dyslipidemia and smoking play major roles in the development of cardiovascular diseases (American Diabetes Association, 2016). Therefore, the impact of depression on macrovascular complications might be more obvious than that on microvascular complications.

In the subgroup analysis, major depression with recurrent episodes had greater associations with macrovascular complications and all-cause mortality than mild depression. These severity-response relationships further support the hypothesis between depression and diabetes complications. In addition, we found that women had a greater risk for mortality and macrovascular complications associated with depression than men. Previous studies have shown that the effect of diabetes on the risk of coronary disease is significantly greater for women than men (Lee *et al*., 2000). Thus, there might be a synergistic effect of depression, female sex and cardiometabolic factors on the risk of diabetes. We found that age carried a multiplicative effect modification. The risk of depression for diabetes complications and mortality was higher among young adults than among middle-aged and older adults. A possible explanation for these findings is that middle-aged or older adults have multiple risk factors for developing complications and mortality; therefore, the effect of depression was not as obvious as that for other risk factors. In contrast, depression might be one of the few risk factors among young adults; depression showed an obvious impact on the development of complications and mortality in our study. The longer duration between diabetes diagnosis and initiation of pharmacotherapy was associated with higher risks for microvascular complications, diabetes-related death and all-cause mortality among patients with depression. Patients with depression have poor lifestyle and difficulty to get benefit from non-pharmacotherapy of diabetes only. These findings might indicate antidiabetic treatment should start early if patients have depressive disorder.

### Strengths and limitations

Several limitations should be considered in this study. First, we identified patients with depressive disorders based on claims records. However, some patients with depression were not diagnosed or treated and could be misclassified into the comparison group; therefore, it would introduce bias towards the null. The effect of depression on diabetes complications and mortality might be underestimated. The duration between the diagnosis of depression and diabetes mellitus was unknown; therefore, we could not explore the effect of duration of depression on the study association. Second, only a few patients with type 1 diabetes mellitus were included because the age of onset was generally before 20 years. Our findings could not be concluded for those with type 1 diabetes mellitus due to the limited statistical power. Third, the accuracy of the diagnosis of microvascular complications of diabetes was not yet validated in the NHIRD; however, we used procedural claims data to confirm ambulatory diagnostic codes, or we only used inpatient claims data to minimise the possibility of misclassification. Fourth, several important factors, such as smoking, body weight, exercise, diet control and family history of depression and/or diabetes are not available in the NHIRD. Although we tried to use chronic pulmonary disease, dyslipidemia, hypertension and alcohol or substance use disorders as proxy measures for smoking, obesity and unhealthy lifestyle behaviours, there were still residual confounding effects. Especially, the lifestyle behaviours are time-variant and would be influenced by depression; therefore, these factors need to be adjusted by advanced methodology (Fewell *et al*., 2004). There is a genetic overlap between depression and type 2 diabetes (Kan *et al*., 2016). The role of the shared genetic architecture on the associations between depression and diabetes complications needs further exploration. Finally, we did not investigate the treatment of depression. The antidepressant exposure might vary and interact with depression severity over the follow-up period; therefore, the association between antidepressant and diabetes complications could not be explored accurately using the current study design. The effect of antidepressant treatment on diabetes complications and mortality warrants further investigations.

The strengths of this study are the novelty of analysing the associations between depression and diabetes complications among incident patients with diabetes, the elimination of confounding by duration of illness and pre-existing complications, the use of a nationwide representative cohort with clear temporal relationships, a very large sample and a well-defined method for identifying complications of diabetes.

## Conclusions

In conclusions, we found that patients with diabetes mellitus had a higher rate of macrovascular complications and all-cause mortality when they had comorbid depressive disorders. However, we found a lower adverse effect of depression than the findings of previous studies. This might be owing to the inclusion in our study sample of only incident patients with diabetes, thereby eliminating confounding owing to the duration of illness and preexisting complications. Depression is a modifiable risk factor of diabetes outcome. Further research should focus on evaluating the effect of depression treatment on advanced complications and mortality among patients with diabetes mellitus.

## Availability of data and materials

The NHIRD used in this study is held by the Taiwan Ministry of Health and Welfare. Any researcher interested in accessing the data set can submit an application form to the Ministry of Health and Welfare requesting access, and we are not permitted to share the data.
